# Strain Tracking to Identify Individualized Patterns of Microbial Strain Stability in the Developing Infant Gut Ecosystem

**DOI:** 10.3389/fped.2020.549844

**Published:** 2020-09-30

**Authors:** Hyunmin Koo, David K. Crossman, Casey D. Morrow

**Affiliations:** ^1^Department of Genetics, University of Alabama at Birmingham, Birmingham, AL, United States; ^2^Department of Cell, Developmental and Integrative Biology, University of Alabama at Birmingham, Birmingham, AL, United States

**Keywords:** transendoscopic enteral tubing, fecal microbiota transplantation, washed microbiota transplantation, pediatric, colonoscopy

## Abstract

Stable microbe and host interactions are established during the development of the infant gut microbial community that provide essential functions for the efficient digestion of food, immune development, and resistance to colonization with pathogens. To further delineate the stability of the gut microbial community during this time, we have used microbial strain tracking analysis with published longitudinal metagenomic data sets to identify strains that persist in the developing infant gut ecosystem. In the first study, 17 infants were evaluated that had not received antibiotics for 3 years after birth. An infant specific pattern was seen for stable and unstable microbial strains during this time, with only one infant having no stable strains identified out of available strains during the first 3 years. Strain tracking was also applied to follow microbes in a separate set of 14 infants that had multiple doses of antibiotics over the 3 years. In 10 out of 14 infants given multiple antibiotics during the first 3 years, we identified a unique pattern of transient strains that appeared after multiple antibiotic treatments for a short time compared to that in infants not on antibiotics. In a second, independent study, we selected a subset of 9 infants from a previously published study consisting of high-density longitudinal fecal sampling to analyze the gut microbial strain stability of *Bacteroides vulgatus* and *Bifidobacterium adolescentis* for up to 6 years following birth. Individual specific patterns were found consisting of varying dominant microbial strains that were independent of antibiotic exposure and birth mode. Our analysis demonstrates an individual specific inherent variability of extinction and persistence of microbial strains in the infant gut community during a time of development that is critical for interactions necessary for establishing normal metabolism and the development of the host immune response.

## Introduction

Previous studies have described the development of the gut microbial ecosystem (1–3). During this time, short-term changes in microbial composition in the infant gut microbiome eventually resolve to a stable microbial composition (4–6). The timing of these changes can be influenced by individual differences (e.g., length of breastfeeding, initiation of solid food), infections that necessitate the use of antibiotics and even exposure to environmental microbial communities (3). In general, the early gut microbial community is dominated by microbes that can feed on the carbohydrates present in breast milk or formula such as *Bifidobacterium adolescentis* (3, 7). As the infant grows, the transition to solid foods and physical growth results in changes in the spatial structure of the gut that contributes to the variation in the physical and chemical environment that provides new ecological niche opportunities for growth of microbial strains (8). This ecosystem transition correlates with the appearance of *Bacteroidetes* (such as *Bacteroides vulgatus*) within the gut microbial community structure.

The establishment of a stable gut microbial community in the developing infant is paramount for microbe-microbe and microbe-host interactions that are needed for essential functions involved in the digestion of food, host metabolism and colonization resistance to pathogens (9, 10). In addition, interactions between the commensal microbes with the host during this time frame are critical for the development of the immune system (7, 11–13). Perturbation of the gut microbial ecosystem from the use of antibiotics during this time has been associated with the development of certain immune dysfunction diseases at later times in children (14). Given the importance of the early years in a life in the development of the functions of the gut microbiome, a more comprehensive understanding of the dynamics of microbial strain stability of the microbial community is essential. In the new era of culture-independent approaches, the use of next-generation sequencing technology has allowed the in-depth examination of microbial genomic variants (i.e., strains) (15, 16). In a previous study, we developed a strain tracking analysis, Window-based single nucleotide variant similarity (WSS), to assess the strain relatedness of multiple microbes in two separate samples (17). Using this analysis, a pairwise genome-wide single nucleotide variant (SNV) similarity comparison can be conducted for a given microbes relatedness between two samples. To distinguish a related strain pair (both strains were taken from the same individual at separate times) from a non-related strain pair (both strains were taken from different individuals), each species' WSS cut-off value for relatedness was used that was established from our previous study based on the Human Microbiome Project (HMP) data set consisting of longitudinal fecal samples taken at times up to 1 year apart (15, 17).

Several recent studies have employed metagenomic sequencing technology to analyze longitudinal samples of the microbial communities in developing infants (1, 2, 18–21).In the current study, the data set from Yassour et al. (19) was chosen for our analysis because it contained longitudinal fecal samples collected from infants with and without antibiotics for the first 3 years of life (19). A second data set of samples were chosen from The Environmental Determinants of Diabetes in the Young (TEDDY) study (1, 2, 22, 23). For our study, we used a subset of the TEDDY samples that included a unique set of high-density longitudinal sampling of fecal samples up to 6 years. A WSS analysis of these two combined studies reveals the individuality of the temporal development of the infant gut microbiome highlighted by the variability of the stability reflected in the extinction and persistence of gut microbial strains in infants during this early period of development.

## Materials and Methods

### Public Data Sets

We used two publicly available data sets: (1) Yassour et al. (19) and (2) TEDDY study (1, 2, 22, 23) to conduct strain tracking analysis.

For Yassour et al. (19), we selected a total of 31 infant samples that included various fecal sample collection time points shortly after birth (<6 months) and at 1, 2, and 3 years (Supplementary Table 1). Within 31 infants, 17 did not receive antibiotic treatment during the first 3 years. However, 14 infants were given multiple antibiotic treatments (average 11.5 times) during the first 3 years due to the associate disease (mostly otitis media). Different classes of antibiotics, including Aminoglycosides, Cephalosporins, Macrolides, Penicillin, and Sulfonamides were used for each infant. Detailed information for each infant regarding times for antibiotics treatment with type of antibiotics was previously published in the Supplementary Table by Yassour et al. (19). For strain tracking analysis, 14 infants' samples were sorted and combined into pre-treatment (average-value of 3.7 months), and post-treatment (average-values of 10, 20, and 23.5 months) (Supplementary Table 1). These time points (3.7, 10, 20, 23.5, and ~33.9 months) did not exactly match with the time points (<6 months, 1, 2, and ~3 years) used for the non-antibiotics treated group, although these still represent the ranges of ~6 months, 1, 2, and ~3 years. All fecal samples were previously sequenced on the Illumina Hiseq 2500 platform with 101 bp paired-end reads (19).

For the TEDDY group, a subset of 9 infants were selected based on high-density longitudinal sampling for up to 6 years (Supplementary Table 2). Similar to Yassour et al. (19), four time points were firstly selected (~6 month, 1, 2, and ~3 years) for these 9 infants and used for the strain-tracking analysis. To include over 3 years samples for these 9 infants, high-density longitudinal samples were additionally included and compared to the last available time point. Of the 9 infants, 5 were chosen that had not received antibiotics throughout sample collection days. Four infants were chosen that had been given multiple antibiotics including Amoxicillin, Penicillin, and others were used for each infant (average 25 times). Detailed information for each infant regarding times and treatment with type of antibiotics will be made available upon request to NIDDK Central Repository at https://www.niddkrepository.org/studies/teddy. Within 4 infants, 3 infants included fecal samples that were collected post-antibiotics, and 1 infant included fecal sample that was collected ~2 months pre-antibiotics. In addition, 4 of the 9 infants developed type 1 diabetes after the last sample collection time. Additional relevant summarized features of the infants from both data sets are found in Supplementary Table 3. All fecal samples of the TEDDY study were previously sequenced on the Illumina Hiseq 2000 platform with 100 bp paired-end reads.

### Total DNA Sequence Reads and Processing

A total of 4,083,347,489 metagenomic sequencing reads were downloaded from the two public data sets; 2,174,856,343 reads from the Yassour et al. (19), and 1,908,491,146 from the TEDDY study (Supplementary Tables 1, 2). Sequences reads were then filtered to remove low quality reads (sliding window of 50 bases having a QScore <20), short sequences (sequence length <50 bases), and cropped at 100 bp using Trimmomatic (version 0.36) (24). Host reads were also filtered by mapping all sequence reads to hg19 human reference genome using bowtie2 (version 2.3.4.3), with default parameters (25). After quality processes, the resultant sequence reads from two data sets were used for the downstream analyses.

### Strain Tracking Analysis Using WSS

From the Yassour et al. (19) data set, 17 of 31 Infants who did not receive antibiotics treatment during the first 3 years were firstly selected to conduct strain tracking analysis using WSS (19). All pairwise comparisons were performed on each infant's sample (<6 months, 1, and 2 years) compared to the same infant's sample that was collected from the last day of the experiment (3 years). For the remaining 14 infants who treated with multiple antibiotics during the first 3 years, all pairwise comparisons were conducted on each infant's sample (pre-treatment: ~3.7 months, post-treatment: 10, 20, and ~23.5 months) compared to the same infant's sample from the last day of the experiment (~3 years; average-value of 33.9 month).

For the TEDDY study (1, 2, 22, 23), each infant's sample (~6 months, 1, and ~2 years) was also compared to the same infant's sample that was collected at ~3 years. To better understand the dynamics of strain change during infants' development, we have additionally included more additional longitudinal samples for up to 6 years for the same 9 infants (Supplementary Table 2). All pairwise comparisons were then conducted particularly for *B. vulgatus* and *B. adolescentis* on each infant's available samples compared to the same infant's last available sample.

For the WSS analysis, the processed reads were aligned to the 93 microbial reference sequences, which were previously established based on the HMP dataset (15, 17) using the Burrows-Wheeler aligner tool BWA-MEM (version 0.7.13) with the -M option (26). Multi-sample SNVs for each given reference sequence were measured among all samples for each infant using the Genome Analysis Toolkit (GATK; version 3.7) (27). The resultant multi-sample Variant Call Format (VCF) files were then used for pairwise comparisons between every possible pair of samples to calculate their overall genome-wide SNV similarity for each microbial species. Any samples having low sequence coverage (<30%) and low sequence depth (<3.5) against their given reference sequences were excluded from the pairwise comparisons (17, 28, 29). A low coverage window with more than 50% of the bases having a read depth <5 were also ignored when comparing the SNV similarity between sample pairs. After quality-based filtering processes, species that were able to provide the WSS score were only selected from each data set. To determine a related strain pair at different time points for each infant, a WSS score for each species was compared against each species' cut-off value that was previously established in our previous study (related strain pair: WSS score > cut-off; unrelated strain pair: WSS score < cut-off) (17, 30), and visualized using Microsoft Excel (Microsoft, Seattle, WA, USA). Species that did not have a cut-off value were excluded from analysis. Full details of the analysis procedure can be found in Koo et al. (29). All codes implemented in the WSS were deposited and are available at https://github.com/hkoo87/mgSNP_2.

Strain-tracking analysis for *Bacteroides vulgatus* was additionally conducted for selected 3 infants from TEDDY data set using StrainPhlAn using default parameters and with the options “-relaxed_parameter3, -marker_in_clade 0.1” (31). Aligned sequence reads against the set of species-specific marker gene database established in MetaPhlAn (32, 33) were used to build a phylogenetic tree of the strains (31). The resultant phylogenetic tree for *B. vulgatus* was visualized using the neighbor-joining method along with the Maximum Composite Likelihood (34) in MEGA X (35, 36) using default parameters.

### Statistical Analysis

Statistical significance (*P* < 0.05) shown in the main text and figure legends was tested by using one-way ANOVA followed by Tukey's multiple-comparisons *post-hoc* test in R (version 3.5.1).

## Results

### Strain Tracking of Infant Gut Microbes After Birth Up to 3 Years

**Figure 1:**
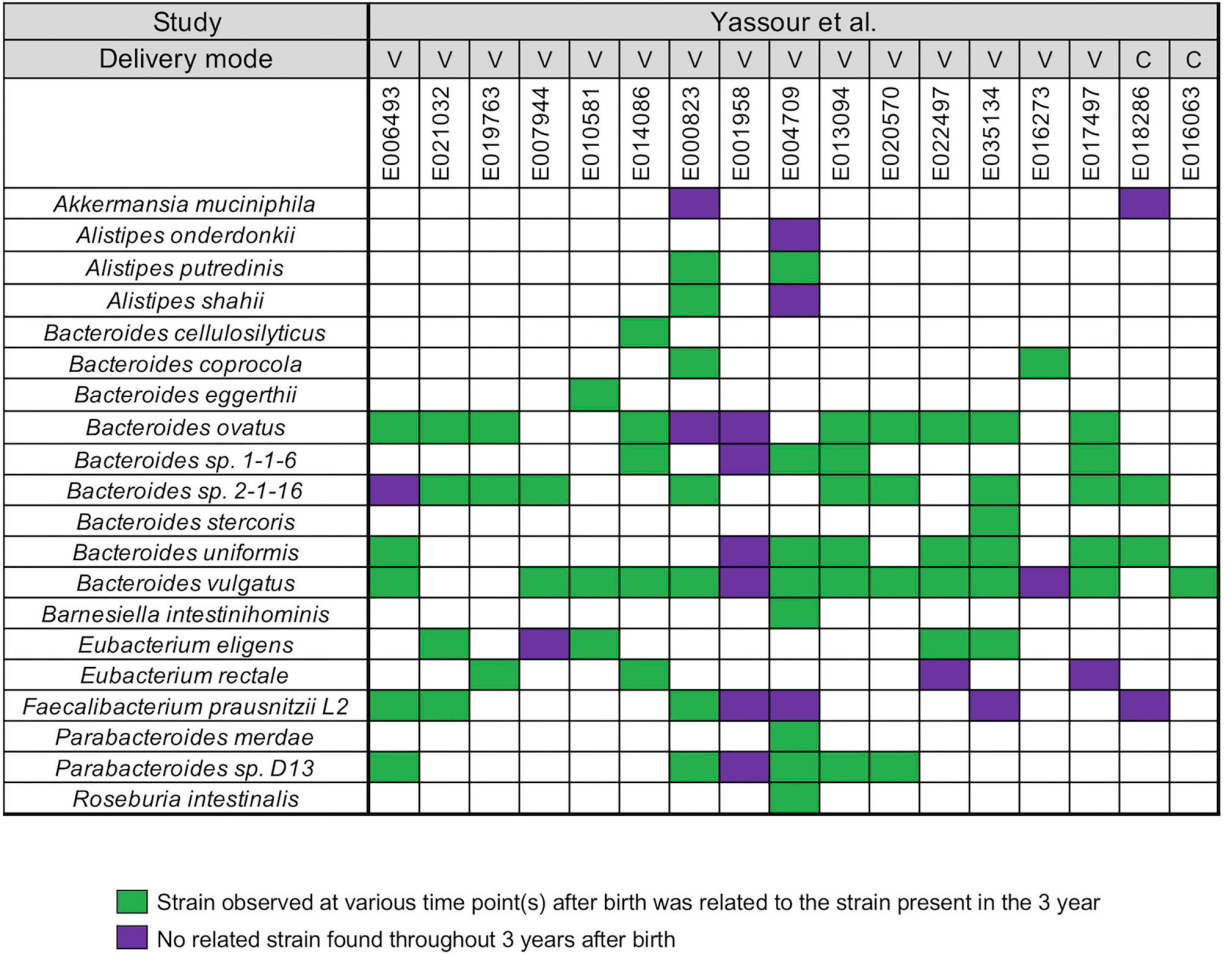
Summarized WSS scores for infants without antibiotic treatment. All pre-processed DNA sequences from Yassour et al. (19) infants without antibiotics were used to compare each infant's last day of the experiment sample (3-year) to every possible pair of the same infant's samples (<6 months, 1-, and 2-year). Twenty species with known WSS cutoff values were identified. The WSS scores for each infant pair that was above the cut-off at some time (<6 months, 1-, and 2-year) compared with the 3 year sample (related) was a green box while samples (<6 months, 1-, and 2-year) compared to 3 years that were all below the cut-off (unrelated) was a purple box. Each column in the table represents an individual's ID and matches the number shown in the Supplementary Table 1. WSS scores for all identified species are provided in the Supplementary Table 4. For delivery mode, V stands for vaginal birth and C for cesarean section birth.

Yassour et al. (19) collected fecal samples from a total 31 infants shortly after birth up to the 3 years (19) (Supplementary Table 1). A WSS analysis was first performed for a subset of 17 infants who did not take antibiotics during this time to determine relatedness of strains during the first 3 years for each infant. After the coverage-based filtering process and excluding any species that did not have a cut-off value, a total of 20 species included members of genera *Akkermansia, Alistipes, Bacteroides, Barnesiella, Eubacterium, Faecalibacterium, Parabacteroides*, and *Roseburia* were found across all infants (Figure 1). For each species, WSS score was obtained from each sample pair and compared against the cut-off value to show the relatedness of the strains (Figure 1). All pairwise comparisons conducted between various time points and the last time point sample for each infant are shown in the Supplementary Table 4. We found that only one infant (E001958) had no related strain pairs appearing during 3 years, although this infant still showed the presence of *Bacteroides, Faecalibacterium*, and *Parabacteroides*, similar to what were observed in the majority of the other infants' microbial communities. In the remaining 16 infants, we noted an infant specific mosaic pattern with dominant strains appearing at different times for some species (green boxes in Figure 1), while for other species, there was no dominant strain detected in 3 years (purple boxes in Figure 1). Collectively, these results establish an infant specific pattern for the appearance and number of dominant strains during the first 3 years.

**Figure 2:**
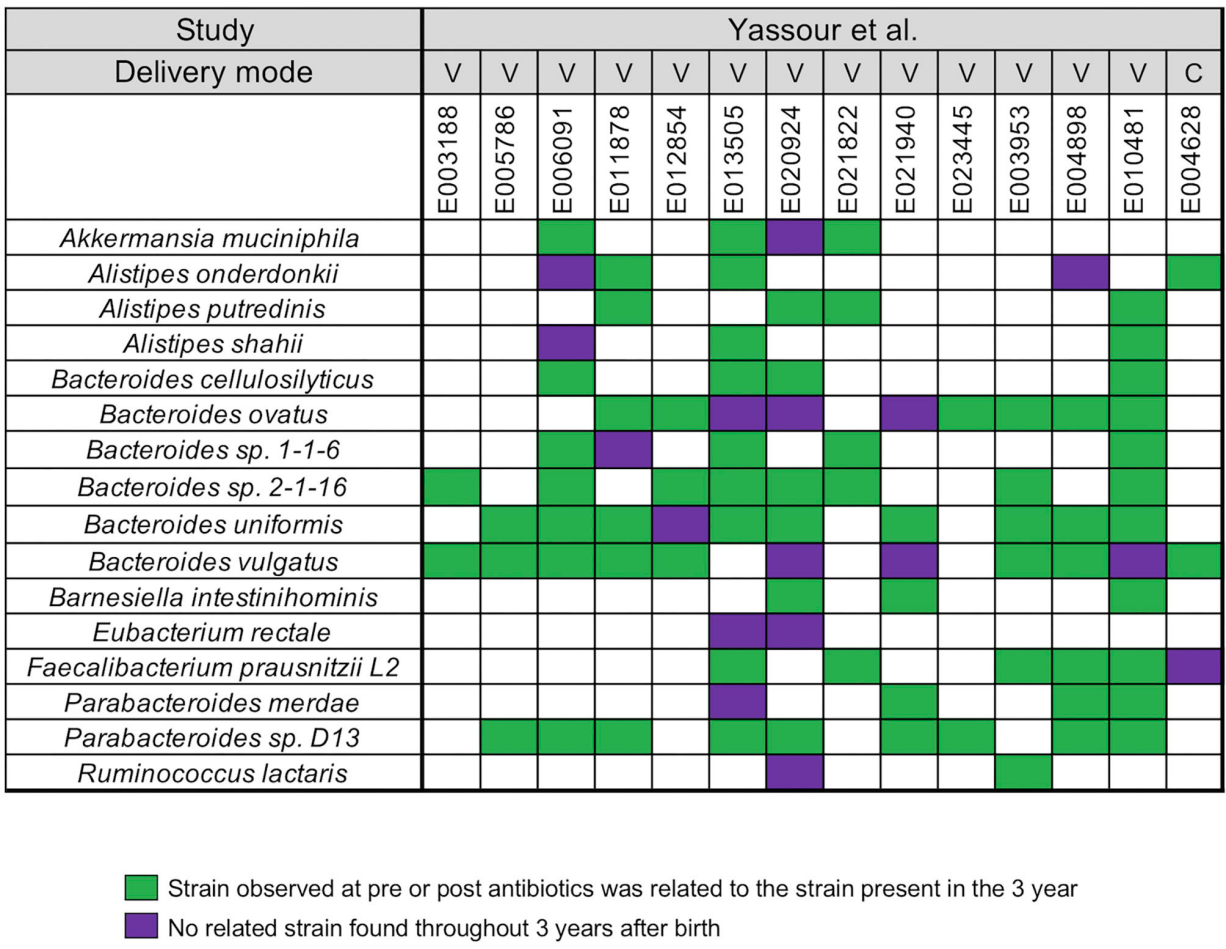
Summarized WSS scores for infants with multiple antibiotics treatments. All pre-processed DNA sequences from Yassour et al. (19) infants with multiple doses of antibiotics over the first 3 years due to ear infection (otitis media) were used to compare each infant's last day of the experiment sample (~3 year; average-value of 33.9 month) to every possible pair of the same infant's samples (pre-treatment: 3.7 months and post-treatment: 10, 20, and 23.5 months). Sixteen species with known WSS cutoff values were identified. The WSS scores for each infant pair that was above the cut-off at some time (pre-treatment: 3.7 months and post-treatment: 10, 20, and 23.5 months) compared with the ~3 year sample (related) was a green box while samples (pre-treatment: 3.7 months and post-treatment: 10, 20, and 23.5 months) compared to ~3 years that were all below the cut-off (unrelated) was a purple box. Each column in the table shows an individual ID and matches the number shown in the Supplementary Table 1. WSS scores for all observed species are provided in the Supplementary Table 5. For delivery mode, V represents vaginal birth and C for cesarean section birth.

In our next analysis, we again used the data set of Yassour et al. (19) to examine the impact of sequential antibiotics on infant's microbial populations during the first 3 years (19). Samples from four time points were selected from 14 antibiotic treated infants, divided into pre-treatment (average-value of 3.7 months) and post-treatment (average-values of 10, 20, and 23.5 months) samples (Supplementary Table 1). For this analysis, we did not stratify the analysis with respect to individual antibiotics or number of antibiotics in between the collection times. A similar array of microbial species, except for *Roseburia*, was identified in the antibiotic treated infants when compared to the infants that did not receive antibiotics (Figure 2). For all infants, we found a unique infant specific pattern that consisted of dominant strain that was stable between the pre- and post-treatment samples up to the 3 years, the limit of the study. All pairwise comparisons conducted between various time points (pre- and post-treatment) and the last time point sample per infant were shown in the Supplementary Table 5.

**Figure 3:**
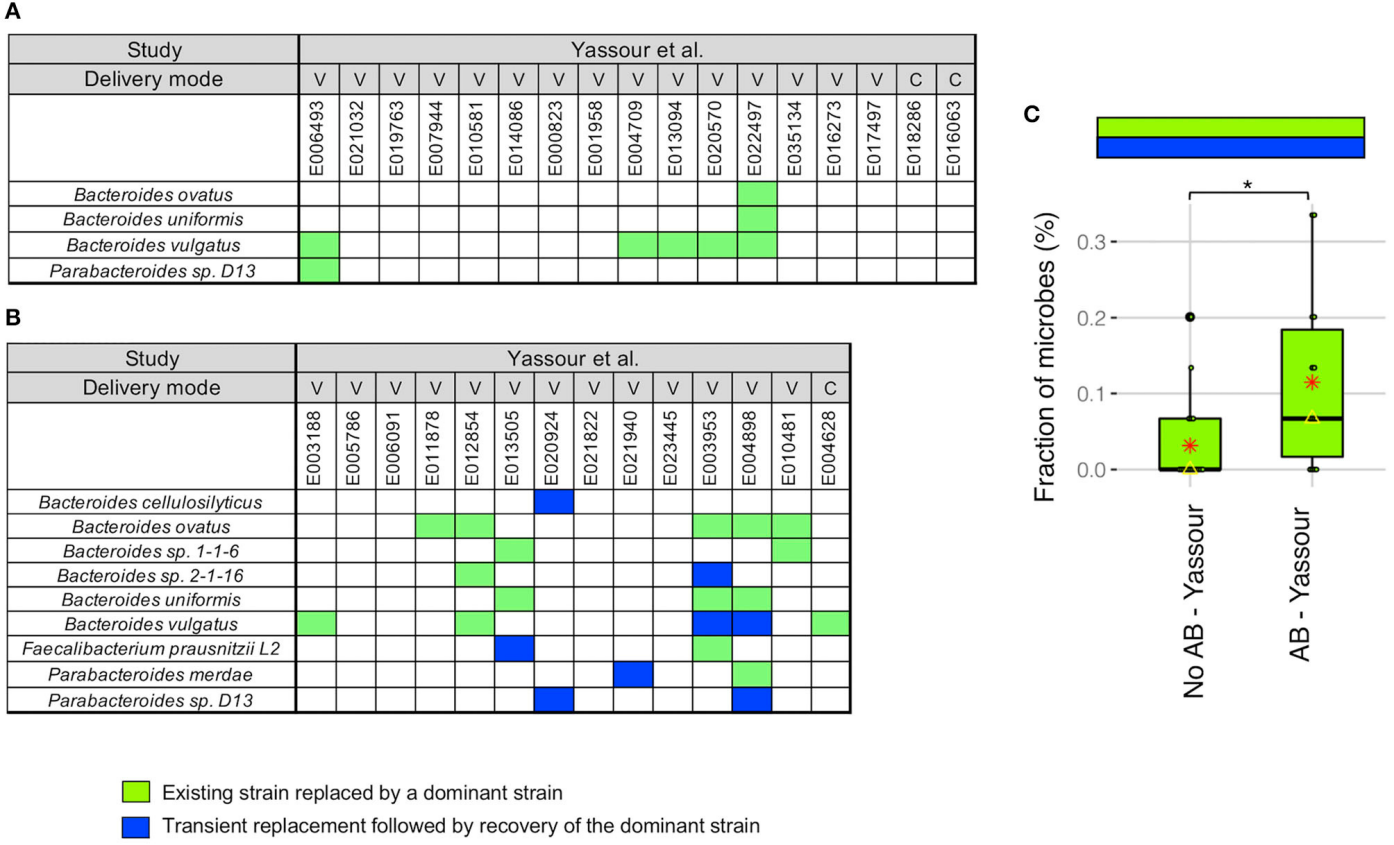
Comparison of strain stability in infants from Yassour et al. with and without antibiotics. A total of 9 species that had WSS cut-off values were used to more detailed examination of the strain stability for infants who **(A)** did not treated with antibiotics and **(B)** did treated with multiple doses of antibiotics over the first 3 years. For each infant, a stability pattern of the related strain (green boxes in Figures 1, 2) during the first 3 years were examined and common patterns for all infants were grouped into two: (1) an existing strain was replaced by a dominant strain (light green boxes), and (2) transient replacement of the dominant strain followed by the subsequent recovery of the dominant strain (blue boxes). For delivery mode, V represents vaginal birth and C for cesarean section birth. **(C)** Significant differences (*P* < 0.05) of the strain stability between with and without antibiotics treatment were tested using an ANOVA followed by Tukey's multiple-comparisons *post-hoc* tests in R (version 3.5.1). *P < 0.05.

We next did a more detailed examination of the strain stability in the Yassour et al. (19) study. For infants without antibiotics treatment, we identified a unique pattern in 5 infants out of 17 where the existing strain was replaced by a new strain that was then dominant up to 3 years (light green boxes in Figure 3A). This replacement occurred in 5 of the 6 infants with *B. vulgatus* although no other microbe was detected more than one time. As an example, for *B. vulgatus* for infant E022497, we found that the WSS score obtained by comparing the sample taken shortly after birth (2.3 months) and the 3-year sample (35.7 months) was 8%, while the WSS score obtained from the samples taken at later times compared with the same 3-year sample was both 100% (Supplementary Table 4).

For the infants given antibiotics, 8 of the 14 infants showed the same strain change pattern as that for the no antibiotic treated infants (light green boxes in Figure 3B). However, there were 5 infants where we found a unique pattern where a transient strain appeared for a short time after the treatment and then returning back to the dominant strain by 3 years (blue boxes in Figure 3B). All of these 5 infants were delivered vaginally and given different antibiotic combinations; the time point when the transient strain appeared was varies for these infants (minimum month of 5.7 found in E004898, and maximum month of 24.1 found in E013505; Supplementary Tables 1, 5). Moreover, 2 of 5 infants had transient strain during breast-feeding period, and 3 infants had transient strain after completion of the breast-feeding period. Detailed metadata regarding the type of antibiotics, length of treatment, and diet information for each infant can be found in Yassour et al. (19). Overall, we found a significant increase in the presence of the replacement patterns in the antibiotics treated group as compared to the no antibiotic treated group (Figure 3C).

### Strain Tracking of Infants After Birth Up to 6 Years

To gain more insights into the dynamics of strain change during infants' development, we analyzed an extensive data set from the TEDDY study where longitudinal samples were taken almost at monthly intervals as early as 6 months for up to 6 years for certain subjects (1, 2, 22, 23). We focused our study on representative subjects with a high number of samples over a short time interval; in addition, we filtered out samples that did not have enough sequence read coverage and depth against our reference sequences to provide a WSS score (17, 28, 29).

For each infant, we first analyzed the strain's relationship of selected samples (after birth up to 3 years) to the same infant's sample that was collected at ~3 years (similar to the Yassour et al.) to select the most abundant species across two different data sets. All pairwise comparisons conducted between various time points (~6 months, 1 and ~2 years) and the last time point sample (~3 years) per infant were shown in the Supplementary Table 6. From this analysis, we found an infant specific mosaic pattern with dominant strains appearing at different times for some species, or no dominant strain observed in the first 3 years, which is similar to what we observed in Yassour et al. (19). *B. vulgatus* was the most abundant species and thus focused our extensive longitudinal sample (up to 6 years) comparisons on this species. Additionally, we have included another species, *B. adolescentis*, for comparative analysis since the importance of this species in early childhood was reported by Vatanen et al. (2). All pairwise comparisons conducted between all available time points and the last available time point (up to 6 years) sample per infant were shown in the Supplementary Table 7.

**Figure 4:**
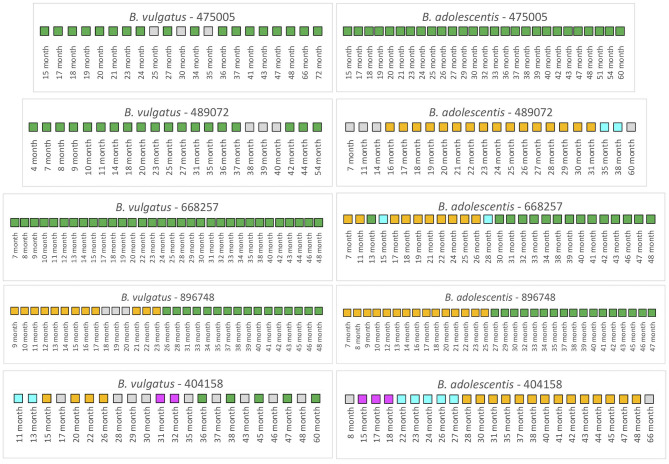
Summarized WSS scores for infants without antibiotics from TEDDY study. The WSS scores were determined comparing the infant's last day of the experiment sample (up to 6 years) to every available pair of the same infant's sample. Note that sample numbers are varied for each infant, and all samples used for the analysis were listed in the Supplementary Table 2. Also, the last date of analyzed samples for four infants (475005, 489072, 896748, and 404158) was different between *Bacteroides vulgatus* and *Bifidobacterium adolescentis* due to not enough sequences detected of that particular species on that sample date. The summarized WSS scores for both *B. vulgatus* and *B. adolescentis* per each infant, not treated with antibiotics, were grouped into different color boxes: (1) Green boxes: observed strain was related to the last available sample (above the respective cut-off value for either *B. vulgatus* or *B. adolescentis*); (2) Yellow, cyan, and magenta boxes: observed strain was not related to the last available sample, but related to other month's samples (related samples have the same color); and (3) Gray boxes: observed strain was not related to any samples (even with the same gray colors). Each individual ID matches the number shown in the Supplementary Tables 2, 3. WSS scores for all pairwise comparisons are provided in the Supplementary Table 7.

We first examined 5 infants from the TEDDY study that had not received antibiotics over the duration of the collection period (Figure 4). For infant 475005, the comparison of the *B. vulgatus* strain at month 72 with the strains at the other collection times revealed considerable stability with only unrelated strains found at months 25 and 35. Similarly, the *B. adolescentis* also exhibited a remarkable stability throughout ~45 months. A similar pattern was found for *B. vulgatus* in infant 489072 from the analysis of samples during 50 months. In this case, all samples were related to the month 54 sample except those obtained from 38, 39, and 40 months, which were below the cut-off. At this time, this subject's *B. vulgatus* strain was not related to the earlier strains or the month 54 strain. We interpret this as a strain variation since by month 42 the *B. vulgatus* was again related to the month 54 strain. The WSS scores from comparison of the months 38, 39, and 40 samples with each other indicated that these samples were not related to each other or any of other samples taken at other times. In contrast, multiple strains of *B. adolescentis* were detected in this subject during 53 months; the strain found at month 31 was related from 16 to 31 months and then a new strain appeared during months 35–38. Examination of the metadata from this subject did not provide an explanation for the strain variation (Supplementary Table 3). Samples collected from infant 668257 from 7 to 48 months were all related indicating there was no strain change of *B. vulgatus* in this subject during 41 months. However, this subject had multiple strains of *B. adolescentis*. The sample collected at month 13 and all samples collected after 30 months were all related to the strain in the last sample (month 48). There were two strains though were detected in this subject, one appeared at 7–11 months and 17–26 months; and another one found at month 15 and 28.

A different pattern was seen for infant 896748. Comparison of the month 48 strain with the other samples' strains revealed a unique pattern where the related strain appeared during 9–17 months, disrupted by other strains during 18–20 months, reappeared during months 21–23, and then replaced with the new dominant strain after month 26. During months 9–17 and 21–23, comparison of these samples with each other reveled they were all related to each other but not the month 48 sample. Interestingly, in this infant we found that the WSS score between two strains of *B. vulgatus*, which were detected at month 23 and month 48 had a score of 20 (Supplementary Table 7). In our previous study on the impact of antibiotics on the strain composition of adults, we found drastic differences in the WSS score between the two strains reflective of a strain replacement (28). Examination of the metadata of this subject at these time points though revealed no definitive reason, such as antibiotic use, that could account for the disruption in the strain stability (Supplementary Table 3). However, we found several additional strains that were not related to the month 23 or month 48, and each other that appeared in this subject (gray boxes in Figure 4).

To substantiate the WSS analysis, we have additionally conducted StrainPhlAn for selected 3 infants (475005, 489072, 896748) to assess strain relatedness between samples per each infant. Since *B. vulgatus* was used for the WSS analysis on TEDDY study, we selected this species to conduct StrainPhlAn (Supplementary Figures 1–3). In this analysis, we found two cases (475005 and 896748) where the WSS and StrainPhlAn agreed (Supplementary Figures 1, 3). StrainPhlAn analysis on the remaining case (489072) represented a partial agreement or disagreement with the WSS with a small difference in branch length (Supplementary Figure 2).

Finally, infant 404158 had the most complex pattern of *B. vulgatus* strains. The last strain (at 60 months) cycled in dominance in this individual related to the strains at 36, 38, 45, and 47 months. The WSS analysis of all sample pairs revealed 3 additional combinations that were above the cut-off value. The first was the month 13 sample that was related with the 11-month; the second was the month 26 sample that was related with the sample at months 15, 20, and 22; and the third was month 31 that was shared with month 32. This extreme instability was also shown in *B. adolescentis*, suggesting the possibility of a system wide instability in the gut ecosystem of this individual that is unique to this individual. Examination of the metadata for this subject could not account for this complex pattern of variation in strains (Supplementary Table 3).

**Figure 5:**
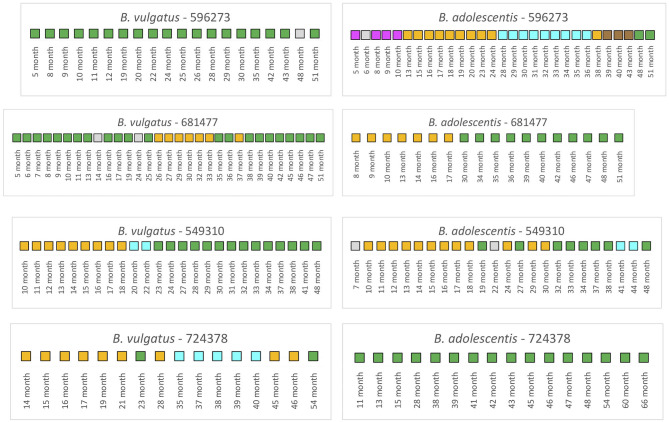
Summarized WSS scores for infants with antibiotics from TEDDY study. The WSS scores were determined comparing the infant's last day of the experiment sample (up to 6 years) to every available pair of the same infant's sample. Note that sample numbers are varied for each infant, and all samples used for the analysis were listed in the Supplementary Table 2. Also, the last date of analyzed samples for one infant (724378) was different between *Bacteroides vulgatus* and *Bifidobacterium adolescentis* due to not enough sequences detected of that particular species on that sample date. The summarized WSS scores for both *B. vulgatus* and *B. adolescentis* per each infant, treated with antibiotics, were grouped into different color boxes: (1) Green boxes: observed strain was related to the last available sample (above the respective cut-off value for either *B. vulgatus* or *B. adolescentis*); (2) Yellow, cyan, magenta, and brown boxes: observed strain was not related to the last available sample, but related to other month's samples (related samples have the same color); and (3) Gray boxes: observed strain was not related to any samples (even with the same gray colors). Each individual ID matches the number shown in the Supplementary Tables 2, 3. WSS scores for all pairwise comparisons are provided in the Supplementary Table 7.

We next examined the TEDDY subjects (*n* = 4) that were treated with antibiotics during the collection periods (Figure 5). Note here that samples that selected for the analysis for 3 of the 4 infants included both pre and post antibiotic treatments samples; only one infant, 549310, included post antibiotic treatment samples.

Analysis of the infant 596273 revealed stable *B. vulgatus* strains at all time periods except for month 48. In contrast, analysis of the *B. adolescentis* revealed extensive instability when samples were compared to the last time point (month 51). Thus, for this subject, there was no link between the stability of *B. vulgatus* and *B. adolescentis*.

A WSS analysis of infant 681477 revealed transient variation in *B. vulgatus* strains where we detected related strains with the last month (51) from months 5 to 13 and months 16 to 19, month 25, months 35 to 36, and months 38 to 51. In contrast, we detected a single strain change in *B. adolescentis* between months 17 and 30 for this subject, highlighting again that changes of *B. vulgatus* and *B. adolescentis* were not necessarily linked in the same individual.

Analysis of infant 549310 revealed multiple *B. vulgatus* strains. The WSS analysis revealed that samples from months 23 to 41 were related to the sample collected a month 48. Before month 23, samples from months 10 to 17 were related to the sample collected at month 18, and month 20 was only related to the month 22. Analysis of the *B. adolescentis* revealed a similar pattern of strain relatedness in which a strain change occurred around months 19–30 and 41–44. Although examination of the metadata did not reveal any disruptions that could account for the changes that occurred between these months in this subject, the analysis is consistent with antibiotic use during this time frame (28).

Finally, we observed a unique pattern for *B. vulgatus* relatedness from examination of infant 724378. In this infant, we found only the 23 month's sample was related to the last collection time sample (54 month). For the other times, we noted the appearance of several different strains that were not related to the strain in the last collection time. We do not know what caused these strain changes, although we know these changes were not caused by antibiotic treatment since the first time for the antibiotic treatment was at month 58 in this infant. In contrast, the analysis after month 58 in the same subject revealed complete *B. adolescentis* strain stability.

## Discussion

In this study, we have used two published data sets combined with our unique strain tracking program to analyze the stability of microbial strains in infants after birth. We demonstrate an infant specific variation of strain stability consistent with a dynamic process of ongoing strain competition for dominance in the infant microbial community. For most infants, these variations resolve by 6 years post birth to a stable infant specific pattern during this critical time of development.

We have previously applied our WSS analysis to examine human gut microbial strain stability in adult (17, 28, 29). Using a publicly available metagenomic data sets from cohabitating twins, we have shown adult twins in the same environment can share microbial strains for decades, suggesting that microbial strains in healthy human adults have the potential to be stable for extended times (29). In contrast, the results from our strain tracking analyses for both infant data sets highlight the individual specific variations in the stability of the infant gut microbial ecosystem. The first data set, Yassour et al. (19) collected samples from infants during the first 3 years (19). Using a strain tracking analysis, we demonstrated the appearance of a stable microbial strain occurs in an infant specific pattern during 3 years; however, some of the infants did not exhibit a stable microbial strain for the entire 3-year period. Our analysis also revealed the presence of unrelated strains in some infants at an earlier time that were eventually replaced by a dominant strain at a later time (light green boxes in Figure 3), suggesting a more complex microbial strain dynamic might be occurring at later times than the 3-year time point used for this study.

To further resolve the dynamics of gut microbial strain competition during development, we made use of a more extensive data set where samples were taken at a shorter time intervals for up to 6 years after birth (1, 2, 22, 23). One of the unique aspects of the TEDDY study was the extensive longitudinal sampling and metagenomic DNA sequencing, that when coupled with our strain tracking analysis provided a new perspective on the dynamics of gut microbial strain variability. Here, we found an individualized pattern for the development of stable microbial strains as typified by that for *B. vulgatus* and *B. adolescentis*. There are several important points from our analysis. First, there was no obvious linkage between strain changes in *B. vulgatus* and *B. adolescentis*. We found one infant given multiple antibiotics had limited change in *B. vulgatus* strains (e.g., infant 596273) with the extensive change in *B. adolescentis*, while another infant given multiple antibiotics had the opposite pattern of *B. adolescentis* strain change (infant 724378). Second, we also noted several examples of transient microbial strain change without antibiotics treatment for short periods followed by recovery to the dominant strain (notably for *B. vulgatus* in infant 475005 and infant 489072). However, as compared to the infant 475005, a similar pattern was not observed in infant 896748 although these two infants were born with the same delivery mode and completed breastfeeding by ~6 months. We do not know what is the driving force for the transient changes, however we note that these infants were also not on antibiotics during the time of the study that could affect strain change as in adults (28). Finally, we noted several instances of an event that resulted in infant specific complete strain change for *B. vulgatus* (infant 896748) and for *B. adolescentis* (infant 896748). Inspection of the metadata for selected infants revealed no obvious correlation between sex, country of origin, delivery mode, or whether the infant would go onto develop diabetes (Supplementary Table 3). Most probably then, the strain persistence or extinction was a result of uncontrolled differences in individual diets and/or physiological growth that would result in differences in the gut ecosystem that impact the competition between strains for dominance. Consistent with this idea is that disruption of the spatial structure of the adult gut environment can also lead to the appearance of new microbial strains in some individuals, as shown from our previous studies of individuals following surgical disruption of the gastrointestinal tract (30). Collectively, our results highlight that individualized responses result in unique patterns of microbial strain stability.

Numerous studies have shown that antibiotics cause a disruption of the gut microbial composition with a long-term impact on the community structure (37–39). In a recent study, we have used our WSS strain tracking analysis to show that antibiotics can also impact the gut microbial strain stability in adults that we correlated with alterations in the “stability landscape” profile of the microbial community (28, 40). In the current study, we found that the strain tracking analysis of the Yassour et al. data set revealed that antibiotics had resulted in a unique strain change pattern for certain infants (19). We found instances in 5 of the 14 infants that had antibiotics some evidence of transient strain as a result of antibiotics compared to infants not given antibiotics. Although those infants with antibiotics had more transient strain change, the dominant strain did recover in all instances. The analysis of the TEDDY study also revealed the transient disruption of stable microbial strains, particularly for *B. vulgatus* and *B. adolescentis* up to 6 years, although these variations could not fully explain by the type of antibiotics or time of the treatment (1, 2, 28). However, the high-density longitudinal sampling of the TEDDY study did provide us with a detailed picture of the dynamics of the variations of microbial strain change in individual infants. For example, we found several instances where the stable strain was disrupted for a time before recovery back to the original strain: *B. vulgatus* (infants 596273 (48–51 months) or 681477 (mainly 26–38 months) or *B. adolescentis* (infant 549310; 19–32 months). However, these strain changes were not unique to infants given antibiotics as we observed similar patterns in infants that did not receive antibiotics (*B. vulgatus* 475005 and 489072; *B. adolescentis* 668257). The high-density sampling of the TEDDY study also allowed us to identify examples of a more drastic strain change that occurred in infants both without (*B. vulgatus* and *B. adolescentis* infant 896748) and with antibiotics (*B. vulgatus* and *B. adolescentis* infant 681477).

At present, there is a limitation for the application of our WSS approach to study strain change in infants at times points <6 months after birth. Since the 93 reference genomes used in the WSS analysis were selected based on their predominance in healthy adults from the HMP data set (15), the reference genomes did not include some of the microbes such as *Bifidobacterium breve, Bifidobacterium bifidum*, and *Bifidobacterium longum subsp. Infantis* etc. that are present during the first 6 months of life. Our reference genomes did include other *Bifidobacterium* species such as *Bifidobacterium adolescentis L2-32* and *Bifidobacterium longum subsp. longum ATCC 55813* and *B. adolescentis L2-32* that was previously isolated from feces from an infant and the assembled reference genome of this species was deposited to NCBI under accession number PRJNA18197. Even though WSS analysis was conducted for B. *longum subsp. longum ATCC 55813*, the relatedness of those strains to each other over time cannot be investigated at this time due to insufficient numbers from paired longitudinal samples from infants (such as those from the HMP data set) that are needed to establish a WSS cut-off value for related pairs (17).

Finally, a hallmark of the healthy adult microbial community is a stable microbial strain population as described by Shaw et al. as a stability landscape (40). However, the results of our analysis using both data sets highlight that microbial strain change is inherent in developing the infant gut microbial ecosystem. Although we do not know the source of these new microbial strains, our recent study has provided evidence that these strains could be maternal in origin (41). What might be the consequences of a change in the stability of the microbial strains in these infants? The establishment of a stable gut ecosystem is probably important to facilitate microbe-microbe and microbe-host interactions necessary for host metabolic functions and the establishment of colonization resistance (8). For example, recent studies have reported a correlation with the use of antibiotics in infants with the increased possibility for the development of diseases of aberrant immune regulation such as allergies and inflammatory bowel disease (42). Collectively, the results from our current study support a personalized approach consisting of monitoring individual infant gut ecosystems using microbial strain tracking analysis to identify specific strain persistence and extinction that could be correlated with the later development of metabolic and immunologic abnormalities.

## Data Availability Statement

All datasets generated for this study are included in the article/Supplementary Material.

## Author Contributions

HK and CM conceived the study and wrote the manuscript. HK did the bioinformatics analyses on NGS data. DC contributed and commented in the preparation of the manuscript. All authors read and approved the final manuscript.

## Conflict of Interest

The authors declare that the research was conducted in the absence of any commercial or financial relationships that could be construed as a potential conflict of interest.

## Supplementary Material

Supplementary Figure 1 StrainPhlAn on 475005 from TEDDY data set. Strain profiling was conducted for *Bacteroides vulgatus* using StrainPhlAn across all samples in infant 475005. A neighbor-joining (NJ) tree was constructed and the tree is drawn to scale with branch lengths (next to the branches). The distances were then computed using the Maximum Composite Likelihood method (34) and are in the units of the number of base substitutions per site using MEGA X (35, 36). The color boxes shown next to the NJ tree match the color boxes shown in Figure 4.

Supplementary Figure 2 StrainPhlAn on 489072 from TEDDY data set. Strain profiling was performed for *Bacteroides vulgatus* using StrainPhlAn across all samples in infant 489072. A neighbor-joining (NJ) tree was constructed and the tree is drawn to scale with branch lengths (next to the branches). The distances were then computed using the Maximum Composite Likelihood method (34) and are in the units of the number of base substitutions per site using MEGA X (35, 36). The color boxes shown next to the NJ tree match the color boxes shown in Figure 4.

Supplementary Figure 3 StrainPhlAn on 896748 from TEDDY data set. Strain profiling was performed for *Bacteroides vulgatus* using StrainPhlAn across all samples in infant 896748. A neighbor-joining (NJ) tree was constructed and the tree is drawn to scale with branch lengths (next to the branches). The distances were then computed using the Maximum Composite Likelihood method (34) and are in the units of the number of base substitutions per site using MEGA X (35, 36). The color boxes shown next to the NJ tree match the color boxes shown in Figure 4.

Supplementary Table 1 Sequence read information from infant samples **(A)** without and **(B)** with antibiotics treatment. The original sequence files were preprocessed, and deposited by Yassour et al. (19). To conduct the WSS strain tracking analysis, all files were downloaded from NCBI that were collected under BioProject PRJNA290381. The table represents the sequence reads after quality checking and additional filtering.

Supplementary Table 2 Sequence read information from infant samples with and without antibiotics treatment from TEDDY study. To perform the WSS strain tracking analysis, all files were downloaded from NCBI Database of Genotypes and Phenotypes (dbGaP) with the primary accession code phs001443.v1.p1, in accordance with the dbGaP controlled-access authorization process.

Supplementary Table 3 Summarized sample information for **(A)** TEDDY and **(B)** Yassour et al. data sets used in this study. Detailed clinical metadata for each infant from TEDDY study is available in the NIDDK Central Repository at https://www.niddkrepository.org/studies/teddy. Detailed metadata for each infant from Yassour et al. (19) data set is available in the Yassour et al. (19).

Supplementary Table 4 WSS results on infants who were not treated with antibiotics from infant samples collected by Yassour et al. All pairwise comparisons were performed on each sample (shortly after birth (~6 months), 1-, and 2-year) compared to the sample from the last day of the experiment (3-year). The resultant WSS scores are represented as a numerical value along with the data bar graphs. WSS scores that were above the cut-off (CO) values are in red. Species that have a CO value were selected and used for comparison analysis. “CO: NA” indicates that there was no CO value assigned to this species, thus excluded to distinguish a related strain pair.

Supplementary Table 5 WSS results on infants treated with antibiotics from Yassour et al. All pairwise comparisons were performed on each sample (pre-treatment: ~3.7 months, post-treatment: 10, 20, and ~23.5 months) compared to the sample from the last day of the experiment (3-years; average-value of 33.9 month). The resultant WSS scores are represented as a numerical value along with the data bar graphs. WSS scores that were above the cut-off (CO) values are in red. Species that have a CO value were selected and used for comparison analysis. “NA” indicates that sample was not available to run the analysis. “NS” represents that no WSS score observed. “CO: NA” indicates that there was no CO value assigned to this species, thus excluded to distinguish a related strain pair.

Supplementary Table 6 WSS results on infants treated with and without antibiotics from TEDDY study. All pairwise comparisons were performed on each sample (~6 month, 1, and ~2 years) compared to the 3-year sample, similar to Yassour et al. (19). The resultant WSS scores are shown as a numerical value along with the data bar graphs. WSS scores that were above the cut-off (CO) values are in red. “NS” represents that no WSS score observed. “CO: NA” indicates that there was no WSS CO value assigned to this species.

Supplementary Table 7 WSS results on infants (treated with and without antibiotics) for longitudinal samples from TEDDY study. All pairwise comparisons were conducted on all available samples (samples are varied for each infant) to the last available sample. If samples did not relate to the last available sample, those were compared with all other samples to observe related strain. WSS scores that were above the cut-off values are in red. Note that the results for *Bacteroides vulgatus* and *Bifidobacterium adolescentis* were presented in Figures 4, 5.
