# Supplementary figures and images for: Strain Tracking to Identify Individualized Patterns of Microbial Strain Stability in the Developing Infant Gut Ecosystem
Source: Front Pediatr. 2020 Sep 30;8:549844. doi: 10.3389/fped.2020.549844 (PMC7555834; doi:10.3389/fped.2020.549844)

Supplementary Figure 1

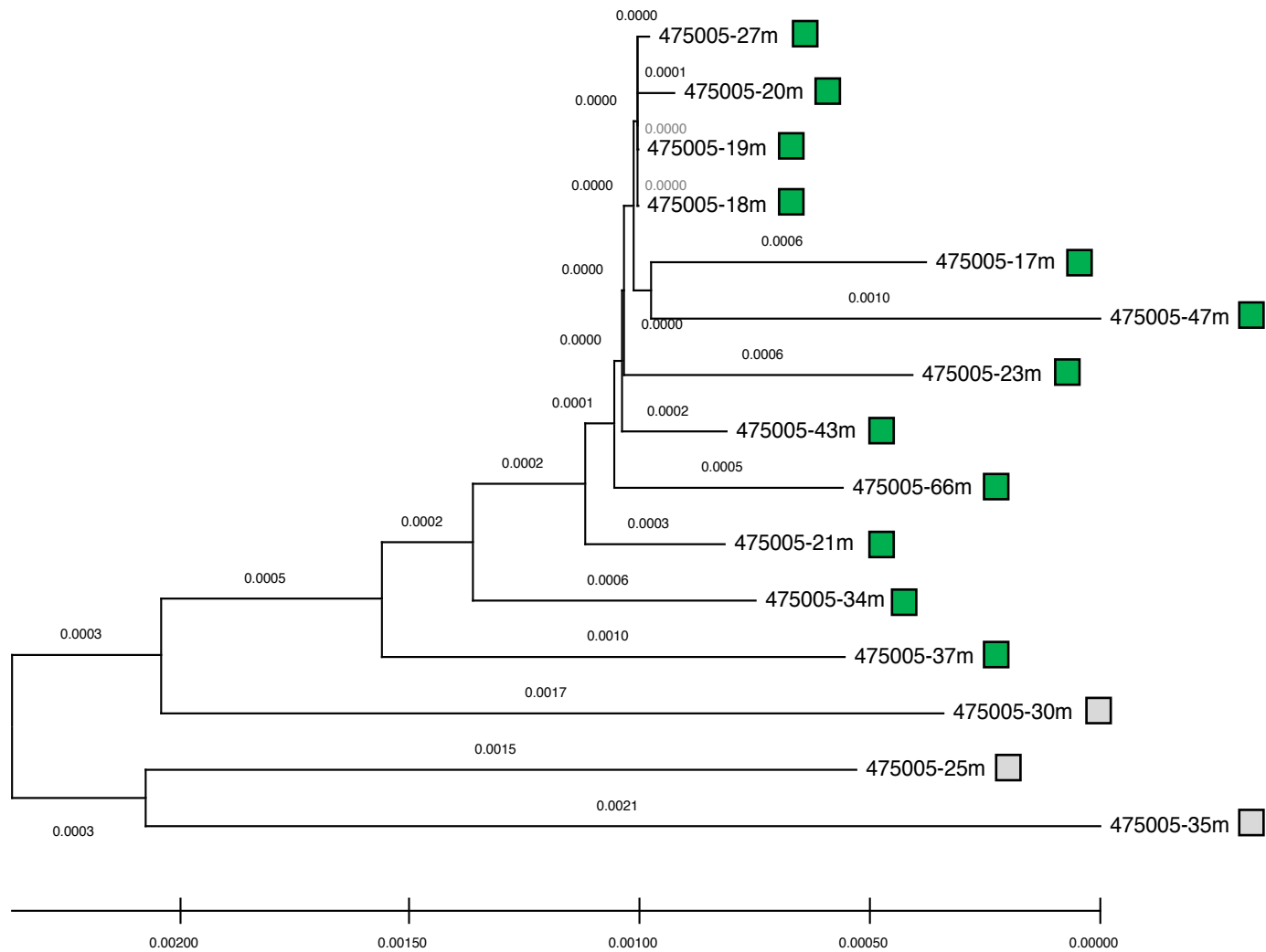

Supplement: Supplementary Figure 1 — StrainPhlAn on 475005 from TEDDY data set. Strain profiling was conducted for Bacteroides vulgatus using StrainPhlAn across all samples in infant 475005. A neighbor-joining (NJ) tree was constructed and the tree is drawn to scale with branch lengths (next to the branches). The distances were then computed using the Maximum Composite Likelihood method (34) and are in the units of the number of base substitutions per site using MEGA X (35, 36). The color boxes shown next to the NJ tree match the color boxes shown in Figure 4. [file Data_Sheet_1.PDF]

Supplementary Figure 2

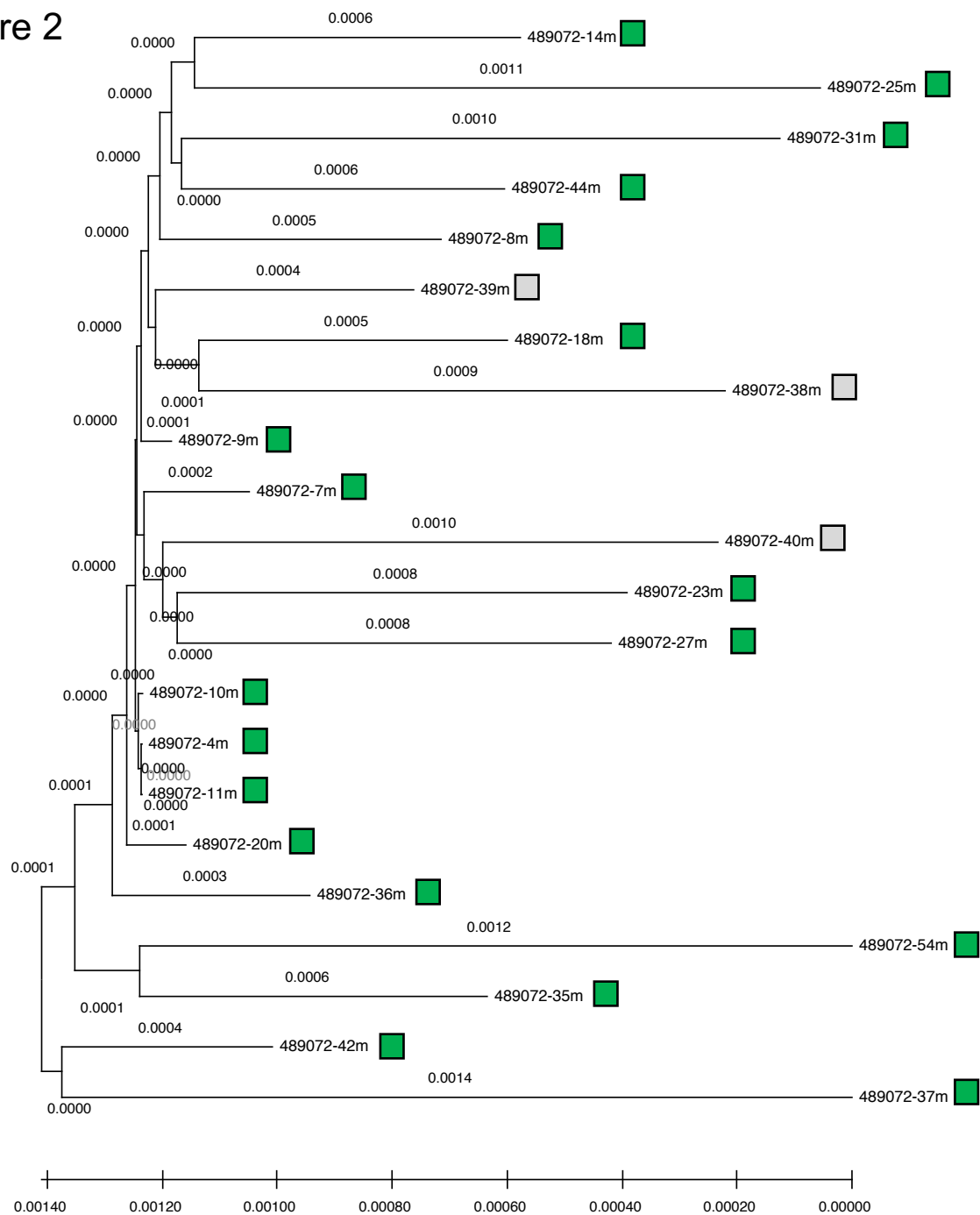

Supplement: Supplementary Figure 2 — StrainPhlAn on 489072 from TEDDY data set. Strain profiling was performed for Bacteroides vulgatus using StrainPhlAn across all samples in infant 489072. A neighbor-joining (NJ) tree was constructed and the tree is drawn to scale with branch lengths (next to the branches). The distances were then computed using the Maximum Composite Likelihood method (34) and are in the units of the number of base substitutions per site using MEGA X (35, 36). The color boxes shown next to the NJ tree match the color boxes shown in Figure 4. [file Data_Sheet_2.PDF]

### Supplementary Figure 3

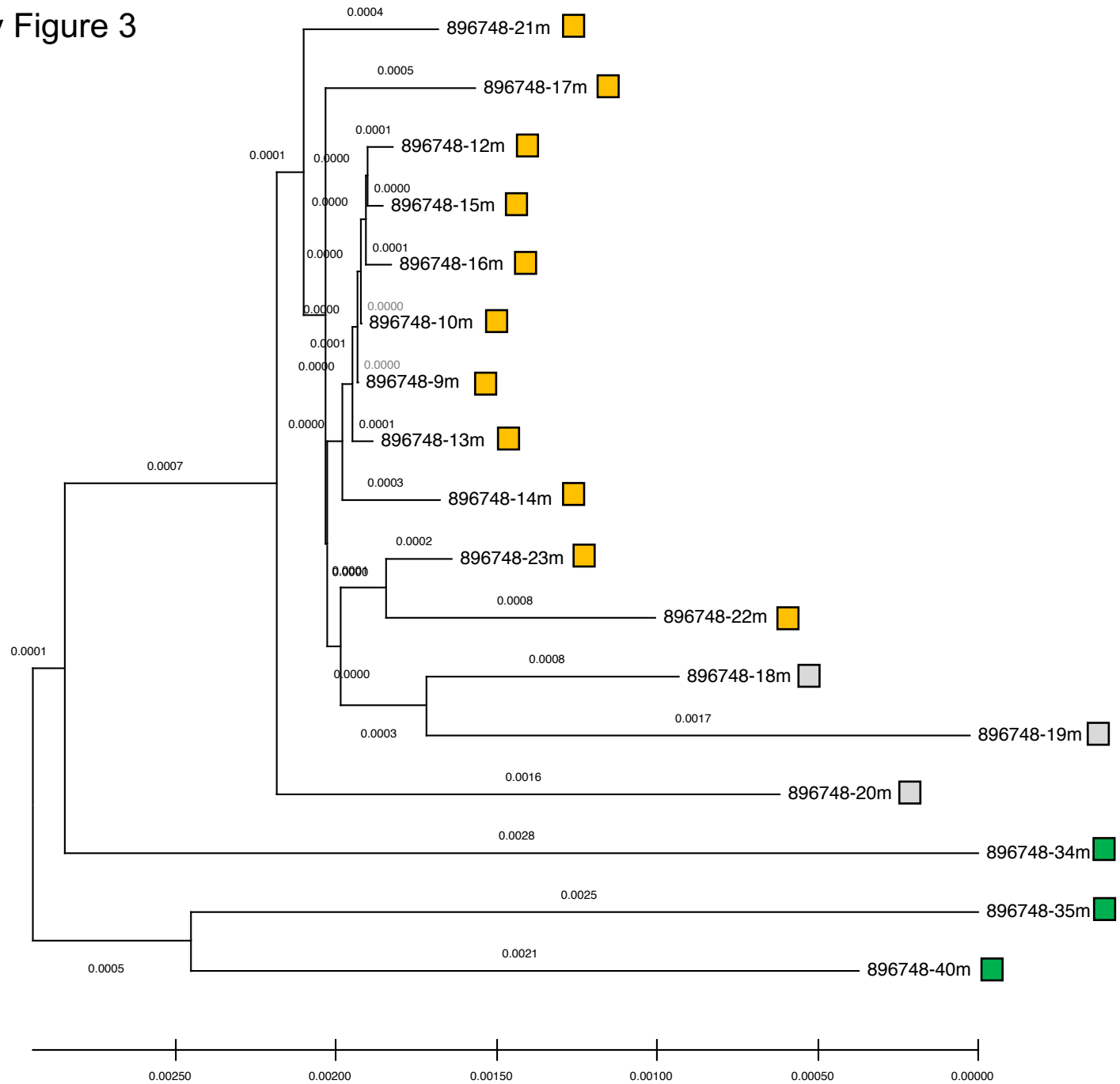

Supplement: Supplementary Figure 3 — StrainPhlAn on 896748 from TEDDY data set. Strain profiling was performed for Bacteroides vulgatus using StrainPhlAn across all samples in infant 896748. A neighbor-joining (NJ) tree was constructed and the tree is drawn to scale with branch lengths (next to the branches). The distances were then computed using the Maximum Composite Likelihood method (34) and are in the units of the number of base substitutions per site using MEGA X (35, 36). The color boxes shown next to the NJ tree match the color boxes shown in Figure 4. [file Data_Sheet_3.PDF]
